# Nickel clusters embedded in carbon nanotubes as high performance magnets

**DOI:** 10.1038/srep15033

**Published:** 2015-10-13

**Authors:** Hidetsugu Shiozawa, Antonio Briones-Leon, Oleg Domanov, Georg Zechner, Yuta Sato, Kazu Suenaga, Takeshi Saito, Michael Eisterer, Eugen Weschke, Wolfgang Lang, Herwig Peterlik, Thomas Pichler

**Affiliations:** 1 Faculty of Physics, University of Vienna, Boltzmanngasse 5, 1090 Vienna, Austria; 2 Nanomaterials Research Institute, AIST, 1-1-1 Higashi, Tsukuba 305-8565, Japan; 3 Atominstitut, TU Wien, Stadionallee 2, 1020 Vienna, Austria; 4 Helmholtz-Zentrum Berlin für Materialien und Energie GmbH, Albert-Einstein-Str. 15, 12489 Berlin, Germany.

## Abstract

Ensembles of fcc nickel nanowires have been synthesized with defined mean sizes in the interior of single-wall carbon nanotubes. The method allows the intrinsic nature of single-domain magnets to emerge with large coercivity as their size becomes as small as the exchange length of nickel. By means of X-ray magnetic circular dichroism we probe electronic interactions at nickel-carbon interfaces where nickel exhibit no hysteresis and size-dependent spin magnetic moment. A manifestation of the interacting two subsystems on a bulk scale is traced in the nanotube’s magnetoresistance as explained within the framework of weak localization.

Magnetic metal clusters continue to attract focus in a broad range of research fields from low dimensional physics^1^,^2^,^3^,^4^,^5^,^6^, chemistry^7^ to biotechnology^8^. Magnetic nanostructures have been proven to impact their electronic conduction. The discovery of giant magnetoresistance in layered heterogeneous magnetic systems is a prime example that led to the horizon of new technology based on magnetic nanostructures in semiconductor industry^9^,^10^. The magnetism of nanoscale magnetic devices such as spin transfer torques are the forefront of research in spintronics^11^. While the fundamental properties of freestanding^1^,^3^ or supported clusters^3^,^4^,^5^ have been sought, even down to atomistic scales^6^, it remains a challenge to encapsulate small magnets in advanced electrical materials and control both the bulk magnetism and magnetotransport properties as desired. Of great importance to this end is the understanding of a causal relationship between changes in magnetic properties on a nanoscale and transport properties on a bulk scale.

In this contribution we report a neat method for magnetic nanostructuring within a single-wall carbon nanotube (SWCNT)^12^,^13^,^14^,^15^,^16^,^17^,^18^, that is an advanced conductor with tunable metallicity^19^,^20^. Ensembles of fcc nickel nano clusters have been produced in designer mean dimensions inside SWCNT bundles. The method allows clusters to be formed *in situ* with dimensions smaller than the exchange length of nickel^21^. In such a condition the intrinsic nature of single-domain magnets and their advanced magnetism with large coercivity can be exploited as well as the size effects evaluated with no environmental factors.

From transmission electron microscopy (TEM), X-ray diffraction and bulk magnetization measurements we first show that the nickel clusters are controlled in size and exhibit superparamagnetism that manifests itself as spin glass-like states at low temperatures whose net magnetization is reduced at most upon cooling without an external magnetic field (zero field cooling). The hysteretic behaviours, i.e. finite coercivity and superparamagnetic blocking temperature, are scaled by the cluster size. The coercivity become as large as ~42.5 mT for the smallest cluster, much larger than around 0.1 mT of bulk nickel^22^. The enhanced coercivity strongly supports the formation of single domain magnets.

X-ray magnetic circular dichroism (XMCD) is an exclusive method that allows orbital and spin magnetic moments of minority elements in compounds and alloys to be evaluated^23^,^24^. We show that the smallest clustered nickel encapsulated in SWCNTs exhibit a net magnetic moment much reduced as compared to its bulk value at low temperatures. Importantly, no hysteretic effects are observed with XMCD which suggests nickel spins interacting strongly with conduction electrons of SWCNTs, provided that the XMCD probes predominantly surface atoms due to the electron mean free path^25^. Indeed, the formation of magnetic clusters inside the tubular structure is found to impact the SWCNT’s electronic conduction as well as magnetotransport properties. It is found that the sheet resistance is increased while the low temperature magnetoresistance, known to be negative due to weak localization, is reduced substantially after the cluster formation. Understanding and endohedral control of the bulk magnetism and magnetotransport properties demonstrated in this study would pave the way towards the production of advanced nanomagnets to be applied in spintronics.

## Results

### Structure

**Figure 1:**
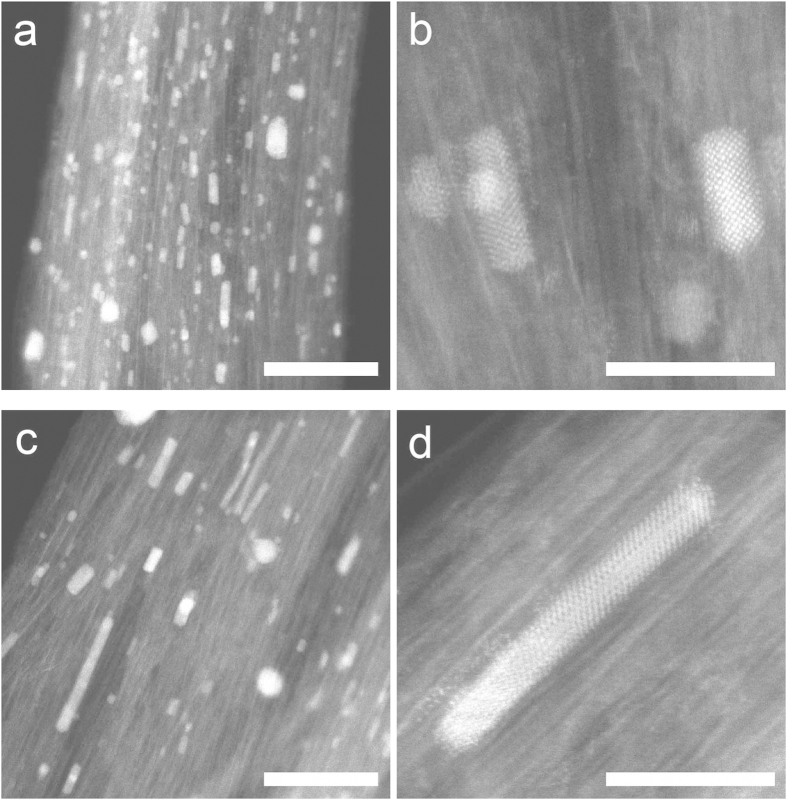
Annular dark field (ADF) scanning transmission electron microscopy (STEM) images of nickel-filled SWCNTs after annealing in vacuum at (a,b) 500 °C and (c,d) 700 °C. Scale bars, 20 nm (**a**,**c**), 5 nm (**b**,**d**).

Figure 1 show annular dark field (ADF) scanning transmission electron microscopy (STEM) images for Ni(II) acetylacetonate-filled SWCNT samples annealed in vacuum at 500 °C for 2 hours (a,b) and 700 °C for 2 hours (c,d). In both cases metallic nickel clusters are observed as bright contrasts inside bundled SWCNTs, see electron energy loss spectroscopy (EELS) data in Supplementary Information (SI). Importantly, there exist less, but longer clusters after annealing at 700 °C, representing that the mean cluster size can be controlled with the annealing temperature. The high-resolution micrographs in panel b and d reveal that the elongated clusters are well crystalline, while the short ones can be amorphous.

### Bulk magnetism

**Figure 2:**
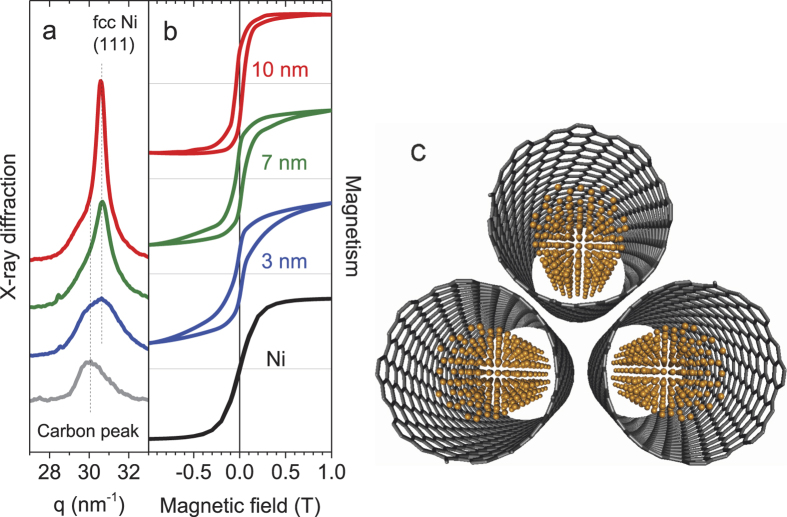
(**a**) X-ray diffraction profiles for three ensembles of nickel clusters in SWCNTs and empty SWCNT. (**b**) The magnetization curves for the 3, 7 and 10 nm clusters in SWCNTs and a bulk nickel measured at 5 K by SQUID. (**c**) Schematic of fcc nickel clusters encapsulated in bundled SWCNTs.

Figure 2b shows the normalized bulk magnetization isotherms measured at 5 K, for nickel clusters encapsulated in SWCNTs with mean cluster sizes of ~3, 7 and 10 nm evaluated from X-ray diffraction measurements. The numerical value has to be seen as a relative measure for the mean cluster size and not an absolute one, as each sample contains nickel clusters with a finite size distribution as observed in the STEM images. See SI for further discussion. No ferromagnetic impurities have been detected in the pristine SWCNTs. The coercive fields are 42.5, 42.2 and 38.5 mT for the 3, 7 and 10 nm clusters, larger for smaller clusters. Importantly, they are significantly larger as compared to less than 1 mT for bulk nickel, suggesting that nickel clusters are of single domain.

**Figure 3:**
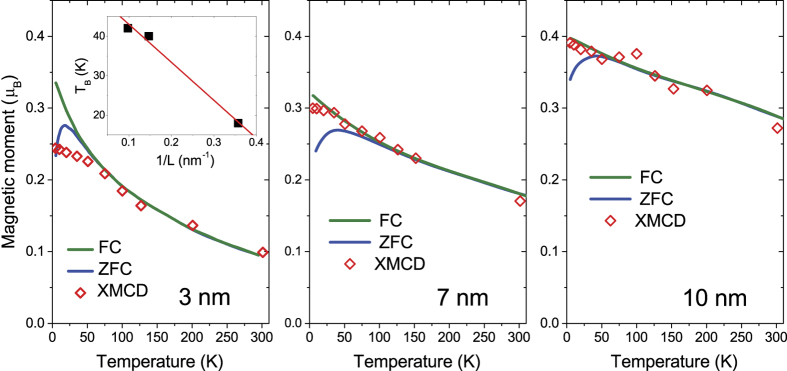
The Ni 3*d* magnetic moments vs temperature measured by XMCD (open rectangles). The bulk magnetization data measured after zero-field (blue curves) and field cooling (green curves) by SQUID and normalized to the XMCD data at high temperatures. Inset: the blocking temperature versus the reciprocal of the cluster length.

The temperature dependence of the magnetization measured in 100 mT, Fig. 3, exhibits hysteretic behaviour upon zero-field (ZFC) and field cooling (FC), characteristic of superparamagnetic materials in which spins of small magnetic domains are frozen to form a spin glass state with a very small net magnetisation upon cooling in zero field while spins are aligned to exhibit a larger net magnetization upon cooling in a finite field. In the former case, the blocking temperature *T*_*B*_ at which the magnetization exhibits a maximum can be defined. We obtain *T*_*B*_ = 18, 40 and 42 K for the 3, 7 and 10 nm clusters that can be inversely scaled by the cluster length as plotted in the Fig. 3a inset^26^,^27^, qualitatively consistent with the previous works on supported ferromagnetic clusters of larger diameters in a range of 2–80 nm^28^,^29^,^30^,^31^.

### Ni 3d orbital and spin magnetism

Encapsulated in SWCNTs, the single domain nickel clusters aren’t affected by environmental factors, allowing these heterogeneous nanostructures to become stable hard magnets. Magnetic properties of each cluster are dominated by the size effect as well as SWCNT-cluster interactions at metal-carbon interfaces. Both together can be accessed exquisitely via XMCD that is intrinsically a surface sensitive method^25^,^32^.

**Figure 4:**
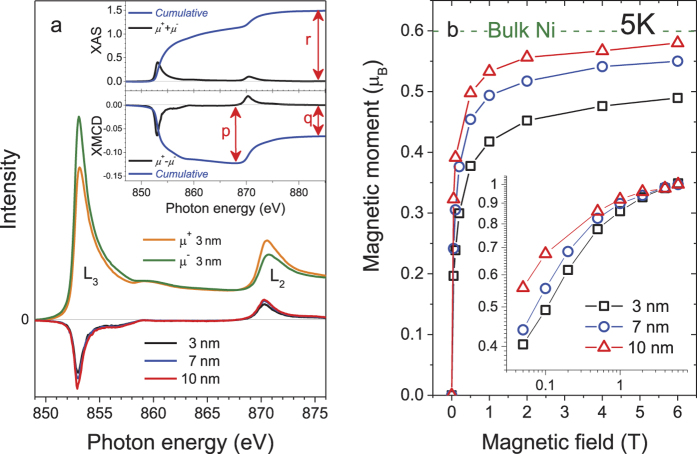
(**a**) Ni 2*p* XAS of the 3 nm Ni cluster and XMCD spectra for the 3, 7 and 10 nm clusters in SWCNTs. Inset: Sum rule parameters, *p, q* and *r*, determined from the cumulative XAS and XMCD spectra. (**b**) The Ni 3*d* magnetization curves for the 3, 7 and 10 nm clusters in SWCNTs measured at 5 K by XMCD.

Figure 4a shows two X-ray absorption (XAS) spectra *μ*^+^ and *μ*^−^ of the 3 nm cluster collected at the Ni L_3,2_ edges with photon spins parallel (+) and anti-parallel (−) to the direction of the magnetic field applied to the sample, respectively. Both Ni L_3,2_ peaks are skewed right and the 6 eV satellite is observed at about 859 eV, characteristic of metallic nickel. The XMCD spectrum is the difference between them, Δ*μ* = *μ*^+^ − *μ*^−^. The orbital 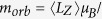
ħ and spin 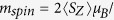
ħ magnetic moments per nickel 3*d* hole can be derived by using the sum rules^33^,^34^,^35^:


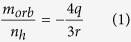


and


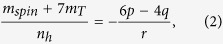


where *n*_*h*_ is the number of holes in the Ni 3*d* states, *p* and *q* are the area intensities of a *μ*^+^ − *μ*^−^ spectrum over the Ni L_3_ and L_2_ edge, respectively, and *r* is the area intensity of a *μ*^+^ + *μ*^−^ spectrum over the Ni L_3,2_ edges, see the Fig. 4a inset. The magnetic dipole term 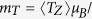
ħ in the spin sum rule, where 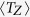 is the expectation value of the magnetic dipole operator, can be omitted^36^,^37^. A degree of circular polarization of 90 ± 2% has been taken into account.

Superposed onto the SQUID data in Fig. 3 are the 3*d* magnetic moments derived from the XMCD data plotted against temperature. The two data sets can be compared since the spin polarization of the Ni 4*s* and 4*p* bands is very small^35^,^38^. Unlike the SQUID data no difference was observed between the ZFC and FC data. The XMCD probes more atoms on the surface than in the bulk because of the mean free path of low energy electrons^25^. This applies even if the cluster diameter is as small as 1–2 nm^32^. In such a case, spin fluctuation as a result of metal spins scattered at the interface by conduction electrons of SWCNTs could influence the degree of the magnetic moment rather than the static spin-glass like disorder which is indeed invisible via XMCD. Note that this fluctuation is more pronounced in the smallest cluster, suggesting the emerging effect of the reduced dimension.

Further in contrast to the SQUID observations (Fig. 2b), the total magnetic moment isotherms estimated from XMCD data vs external magnetic field *H* (Fig. 4b) show no magnetic hysteresis within the limits of experimental error. This indicates that nickel surfaces are much softer than the bulk as if a hard core-soft shell magnetic structure exist within a cluster.

A quantitative analysis of the magnetization curve as a function of both the applied field and temperature is a difficult task due to the large magnetic anisotropy in crystalline nickel as well as grain size-dependent properties, that cannot be reproduced universally by a theoretical model. Yet, as seen in the Fig. 4b inset in which the magnetization data at 5 K normalized to the maxima are plotted logarithmically against the magnetic field, the initial permeability increases monotonically with increasing the cluster size. The same trend was observed in the previous work on one-order larger diameter ferromagnetic particles at room temperature^29^, but our data saturate in a much lower field, suggesting a higher degree of magnetic ordering.

The saturation values for the spin and orbital magnetic moments obtained at 5 K and 6 T can be regarded as the spin and orbital magnetic moments in the ground state provided that variations in magnetic moments are very small below 5 K and above 6 T. As summarized in Table 1, we obtain *m*_*spin*_ ~ 0.41, 0.48 and 0.50 *μ*_*B*_ for the 3, 7 and 10 nm clusters, respectively, while *m*_*orb*_ ~ 0.077, 0.072 and 0.079 *μ*_*B*_. The previously reported values for bulk fcc Ni are 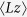 = 0.05–0.06, 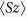 = 0.27–0.29, total moment *m*_*tot*_ = *m*_*orb*_ + *m*_*spin*_ = 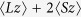 = 0.60–0.62^39^,^40^, Table 1. Obviously, the spin magnetic moments are reduced from the bulk value. The same trend was observed in thin films^40^,^41^, that is in contrast to the case for freestanding nickel clusters in which the magnetic moment increases as the cluster becomes smaller^1^,^42^. This is inline with the temperature dependent data, Fig. 3, and is a clear signature of quenched magnetization at low temperatures as a result of interactions in the solid.

As can be noticed from equation 1 and 2, the ratio between the orbital and spin magnetic moments is independent of the hole number *n*_*h*_, hence provides a more precise measure on the Ni 3*d* ground state when the hole number is not exactly known or could vary. The bulk values reported are *m*_*orb*_/*m*_*spin*_ = 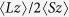 ~ 0.1^34^,^40^,^43^. We have *m*_*orb*_/*m*_*spin*_ ~ 0.19 for the 3 nm cluster which is much larger than ~0.15 and 0.16 for the 7 and 10 nm clusters, respectively. Although the latter values are still larger than ~0.1 for fcc Ni, the much larger value for the smallest cluster is mainly due to the 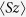 suppressed by up to 25% while the 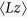 values are equally higher than the bulk value within the limits of experimental error.

Similar observations were made in few layer Ni films with values as high as *m*_*orb*_/*m*_*spin*_ ~ 0.54^41^ or ~0.36–0.39^40^, that could be attributed to the reduced coordination number of surface atoms concomitant with regaining spin-orbital coupling. In our system, only spins are enhanced. There is electron transfer from nickel to carbon that leads to the chemical stability of the metallic nickel^18^. Interactions at the interface could be responsible for the spin suppression, not only those mentioned above.

### Magnetotransport

The magnetism as probed by surface sensitive XMCD should predominantly exist at the interface between the clusters and SWCNTs. If so, all changes caused in the interface Ni spins should be observed via the SWCNT’s conduction electrons in contact. Apparently, the interaction is strongest for the smallest cluster in which the spin polarization is largely reduced at low temperatures, see Table 1. This would naturally impact the electronic conduction of SWCNTs, hence in the following we discuss on their magnetotransport properties.

**Figure 5:**
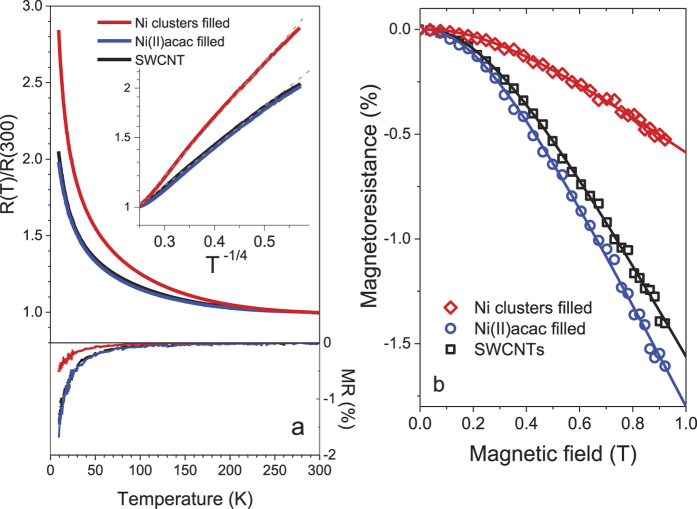
(**a**) Normalized resistance and magnetoresistance (MR) plotted against temperature. In the inset the resistance in logarithmic scale versus *T*^−1/4^ follows the VRH model (dashed grey lines). (**b**) Magnetoresistance vs magnetic field at 9.5 K, fitted to Eq. 3 (solid curves).

The resistance has been measured as a function of temperature without and with magnetic fields up to ~1 T applied to a direction normal to the current direction. The SWCNT’s electronic conduction can be explained within the variable range hopping (VRH) model as the resistance vs temperature *T* follows exp(*T*^−1/(*n *+ 1)^) where *n* = 3 corresponds to the dimensionality, the Fig. 5a inset.

The magnetoresistance is defined by: *M*(*T, B*) = [*R*(*T, B*) − *R*(*T*, 0)]/*R*(*T*, 0), where *R*(*T, B*) is the resistance at temperature *T* and magnetic field *B*, and hence *R*(*T*, 0) in zero field. In low fields *B* < 0.1 T where hysteretic spin glass states emerge at low temperatures, the SWCNT film containing nickel clusters shows only a faint magnetoresistance, which means that the spin disorder among magnetic clusters observed below the blocking temperature in ZFC magnetisation measurements hardly affect the magnetotransport, otherwise alternating direction of cluster spins should give rise to a pronounced negative magnetoresistance if conduction electrons get spin polarized within each magnetic domain. This supports our conclusion that cluster spins interact with conduction electrons only at the metal-carbon interfaces where there is no magnetic order.

Figure 5b shows the field dependence of *M*(*T, B*) measured at 9.5 K. The magnetoresistance stays negative and increases as the field increases. No hysteresis observed in the magnetoresistance upon bipolar field scans within ±1 T support the above-mentioned conclusion that the negative magnetoresistance does not originate from the spin dependent electron scattering, nor the domain wall scattering^44^, since hysteretic features are usually observed near the coercive field.

The field dependence can be fitted well to the following function predicted for a weakly localized three dimensional system^45^:


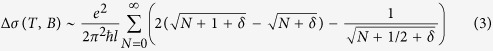


where 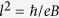 and 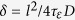, where *D* is diffusion coefficient and *τ*_*ε*_ the energy relaxation time of an electron due to inelastic scattering.

## Discussion

The negative magnetoresistance observed for the SWCNT sheet at low temperatures can be understood as a result of weak localization associated with enhanced backscattering when the elastic scattering dominates the electron conduction, as expected and previously observed for carbon nanotube films in similar field ranges. In higher fields a positive magnetoresistance is known to dictate due to the shrinkage of wave functions within the variable range hopping regime^46^,^47^.

Upon the encapsulation of nickel clusters the negative magnetoresistance is reduced to 37% of the value for the empty SWCNT as the resistance is increased at low temperatures. The fact that more electron-doped SWCNTs^18^, containing paramagnetic Ni(II) acetylacetonate (acac) precursor, exhibit the magnetoresistance very similar to the pristine SWCNT, see Fig. 5, leads us to a conclusion that doping of SWCNTs does not take a major role in reducing the magnetoresistance. Indeed, both electron and hole doping of CNTs reportedly lead to a more negative magnetoresistance^46^. The degree of weak localization can be reduced as a result of an excess phase acquired by a conduction electron via the inclusion of ferromagnetic clusters. This scenario is backed up by the fact that the encapsulation of the paramagnetic Ni(II) acetylacetonate precursor hardly alters the magnetoresistance of SWCNTs.

To conclude, we have demonstrated that the nickel nanomagnets created in SWCNTs outperform their bulky counterparts. With SWCNTs’ advanced properties (high electrical mobility, tuneable energy gap, extremely light weight, flexible and transparent) the method allows nanomagnets to be implemented in various materials and devices. A large change in diffusive conduction of SWCNT webs via the inclusion of nanomagnets indicates much greater impact on the ballistic transport of a single SWCNT that are yet to be studied. A deeper understanding of the magnetic interaction at the metal-carbon interface gained in the present study would help us overcome the superparamagnetic limit in magnetic recording^48^.

## Methods

### Sample preparations

The SWCNT films were filtered out of an ethanol solution of an e-Dips SWCNT material with tube diameters of 1.7 ± 0.1 nm^49^, then annealed in high vacuum at 1300 °C for ca. 18 hours so that no magnetic catalysts were detected afterwards by XRD, SQUID and XPS. Nickel clusters were prepared inside SWCNTs via filling with Ni(II) acetylacetonate, molecular formula C_10_*H*_14_*NiO*_4_, followed by heating in vacuum: The filling process was commenced by heating the SWCNT film in air at 400 °C for 30 minutes and subsequently in vacuum for 2 hours. Then, the SWCNT film was sealed together with Ni(II) acetylacetonate powder (Sigma Aldrich) in an evacuated glass ampule. The sealed ampule was heated at 130 °C for 8 days^12^,^18^, that allows the SWCNT film to be exposed to a vapour of the Ni(II) acetylacetonate. The molecules encapsulated inside SWCNTs can be transformed by heating in vacuum to metal clusters that then act as a catalyst for the formation of inner-shell carbon nanotubes. Hence, whether or not the molecules are encapsulated inside SWCNT can be judged by observing the formation of double-walled carbon nanotubes (DWCNTs). A piece of the film collected from the ampoule was heated in vacuum at temperatures higher than 500 °C, and the radial breathing mode (RBM) lines were observed by Raman spectroscopy, see SI. The cluster’s mean length was controlled by varying the heating temperature and determined from XRD measurements. For XMCD and SQUID measurements three different samples were cut out from one Ni(II) acetylacetonate-filled SWCNT sheet and annealed in vacuum at 500 °C for 2 hours, 700 °C for 2 hours, or 800 °C for 5 hours. For magnetotransport measurements stripes (~2 mm × 10 mm) of SWCNTs with and without filling of nickel, before and after annealing in vacuum at 500° C for 2 hours were prepared. Four point probe contacts were made by pressing gold wires onto the SWCNT stripe.

### Experimental methods

Nickel-filled SWCNT samples were dispersed in n-hexane by sonication and dropped onto molybdenum microgrids covered with holey amorphous carbon films for STEM. ADF-STEM images were obtained by using a JEOL JEM-2100F microscope equipped with Delta spherical aberration correctors at an electron accelerating voltage of 60 kV^50^. Electron energy loss spectroscopy was performed by using a Gatan Quantum spectrometer installed into the microscope.

XRD measurements were carried out at a Bruker Nanostar equipped with a pinhole camera, a 2D gas detector (Vantec 2000) and an image plate (Fuji FLA 7000) for simultaneous measurement of small-angle and wide-angle X-ray scattering patterns. The X-ray patterns were radially averaged to obtain the scattering intensities in dependence on the scattering vector = 4*πsin*(*θ*)/*λ*, where 2*θ* is the scattering angle and *λ* = 0.1542 nm the X-ray wavelength.

The Raman data were recorded at room temperature using a Bruker FT Raman spectrometer with an excitation energy (wavelength) of 1.16 eV (1064 nm). Bulk magnetisation measurements were carried out at Atominstitut, Vienna University of Technology, using a SQUID magnetometer with magnetic fields up to 1 T and temperatures in a range of 5 to 300 K. XMCD measurements were carried out at UE46-PGM-1 variable polarization undulator beamline at BESSY II synchrotron facility, Helmholtz-Zentrum Berlin (HZB). XMCD spectra were recorded by measuring the sample drain current over the Ni 2*p* edge with the photon helicity parallel (*μ*^+^) or antiparallel (*μ*^−^) to the magnetic field applied to the sample. The experimental end-station of this beamline allowed cooling the sample down to 5 K in magnetic fields up to 6 T.

The resistance was measured in four point geometry at temperatures ranging from 9 to 310 K and in external magnetic fields up to 1 T in a setup equipped with a closed-cycle helium cryocooler with the sample attached onto a temperature-controlled cold finger in vacuum. The data were collected with the magnetic field applied normal to the current direction. Switching the magnetic field orientation parallel to the current direction caused no change in the resistance and magnetoresistance. This confirms that the Lorentz force on the carrier doesn’t lead to a significant contribution to the magnetoresistance.

## Additional Information

**How to cite this article**: Shiozawa, H. *et al.* Nickel clusters embedded in carbon nanotubes as high performance magnets. *Sci. Rep.* **5**, 15033; doi: 10.1038/srep15033 (2015).

## Supplementary Material

Supplementary Information

## Figures and Tables

**Table 1:** Magnetic moments *m*_*S*_spin, *m*_*L*_orb, *m*_*tot*_ and their ratio *m*_*L*_orb/*m*_*S*_spin at 5 K and 6 T.

*L*_*p*_/*nm*	*m*_*spin*_/*μ*_*B*_	*m*_*orb*_/*μ*_*B*_	*m*_*tot*_/*μ*_*B*_	*m*_*orb*_/*m*_*spin*_
3	0.41	0.077	0.49	0.19
7	0.48	0.072	0.55	0.15
10	0.50	0.079	0.58	0.16
bulk	0.54–0.57	0.05–0.06	0.60–0.62	0.09–0.1
