# Supplementary material for: Nickel clusters embedded in carbon nanotubes as high performance magnets
Source: Sci Rep. 2015 Oct 13;5:15033. doi: 10.1038/srep15033 (PMC4602218; doi:10.1038/srep15033)
Supplement: Supplementary Information [file srep15033-s1.pdf]

Supplementary Information

Nickel clusters embedded in carbon nanotubes as high performance magnets

Hidetsugu Shiozawa,[1*] Antonio Briones-Leon,[1] Oleg Domanov,[1] Georg Zechner,[1] Yuta Sato,[2] Kazu Suenaga,[2] Takeshi Saito,[2] Michael Eisterer,[3] Eugen Weschke,[4] Wolfgang Lang,[1] Herwig Peterlik,[1] Thomas Pichler,[1]

[1]Faculty of Physics, University of Vienna, Boltzmanngasse 5, 1090 Vienna, Austria,

[2]Nanomaterials Research Institute, AIST, 1-1-1 Higashi, Tsukuba 305-8565, Japan,

[3]Atominstitut, Vienna University of Technology, Stadionallee 2, 1020 Vienna, Austria,

[4]Helmholtz-Zentrum Berlin für Materialien und Energie GmbH, Albert-Einstein-Str. 15, 12489 Berlin, Germany

*To whom correspondence should be addressed; E-mail: hidetsugu.shiozawa@univie.ac.at.

Scanning transmission electron microscopy and electron energy loss spectroscopy analysis

Nickel clusters grown inside SWCNTs by annealing at 500 °C for 2 h were characterized by means of electron energy loss spectroscopy (EELS). EELS data obtained from nickel clusters A-C in Fig. 1a (the same micrograph as Fig. 1a) and that from commercially available nickel (II) oxide (Alfa Aesar, purity 99.998%) are shown in Fig. 1b. Only the nickel $L_{2,3}$ edge is found at around 854 eV in the spectra A, B and C, and the absence of oxygen K edge at around 532 eV indicates that the amount of oxygen in these clusters are negligible. Note that cluster C is presumed to be outside SWCNTs based on its large size. Fig. 2 shows that inside a SWCNT a nickel cluster and an inner carbon nanotube are arranged in tandem. Note that the nickel cluster

is not encapsulated inside the inner tube, but in contact with the outer tube.

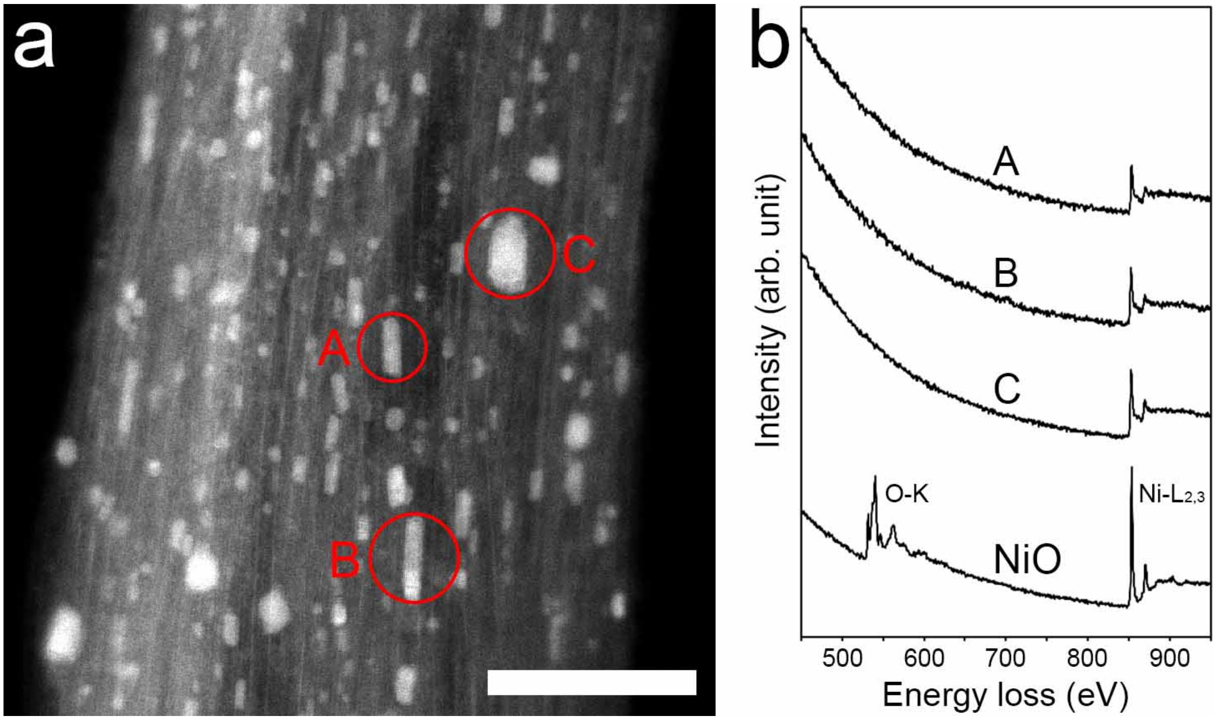

Figure S 1: **a**. *Annular dark field (ADF) scanning transmission electron microscopy (STEM) image of SWCNTs encapsulating nickel clusters after annealing at 500 °C for 2 h. Scale bar, 20 nm.* **b**. *EELS spectra of nickel clusters A-C in a and nickel (II) oxide.*

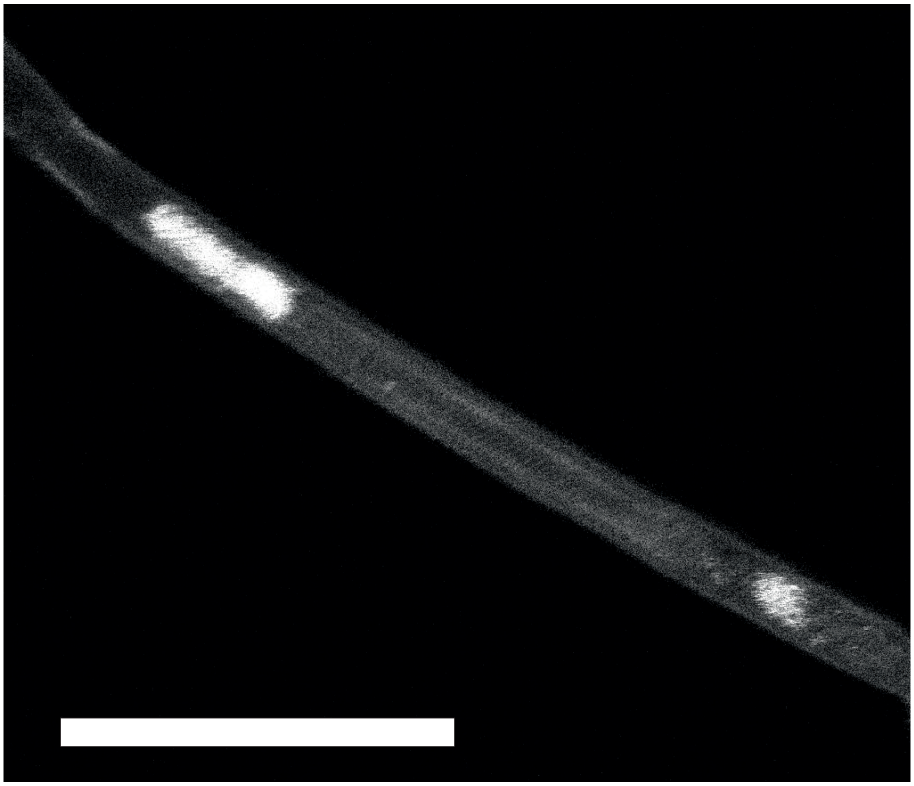

Figure S2: *ADF-STEM image of a SWCNT encapsulating a nickel cluster and an inner carbon nanotube, after annealing at 500 °C for 2 h. Scale bar, 10 nm.*

X-ray diffraction analysis

The diffraction patterns show that inside SWCNTs nickel clusters crystallize in the face-centered cubic structure (fcc) (*1*). The XRD profile of nickel filled SWCNTs exhibits sharp peaks located at 30.6 and 35.4 nm^{-1} assigned to the 111 and 200 reflections of fcc nickel, respectively, Fig. S3. We estimate the effective average size of the fcc nickel clusters from the width of the Ni 111 peak using the Scherrer equation:

$$L = \frac{2\pi K}{\Delta q}, \tag{S1}$$

where Δq is the full width at half maximum and K is the shape factor that was set to 0.9.

The full width at half maximum (FWHM) values for samples annealed at 500 °C for 2 hours, 700 °C for 2 hours and 800 °C for 5 hours are $\Delta q = 2.00$, 0.84 and 0.56 nm^{-1}, which correspond to cluster sizes in length of $L \sim 3$, 7 and 10 nm, respectively. Similar values are obtained from the Ni 200 peaks. This has to be seen as a relative and not an absolute size of the clusters. The absolute value of the cluster size depends on the choice of the Scherrer constant K in Eq. S1, which is dependent on the shape and size distribution of the clusters (*2*). As the precise shape and size distribution is not known in our case, we decided to use the same constant $K = 0.9$ for all curves. As K remains in the order of 1 for all important cases such as spherical, plate-like or rod-like clusters (*3*), we see this as a reasonable compromise to describe the relative increase of the cluster size with annealing temperature.

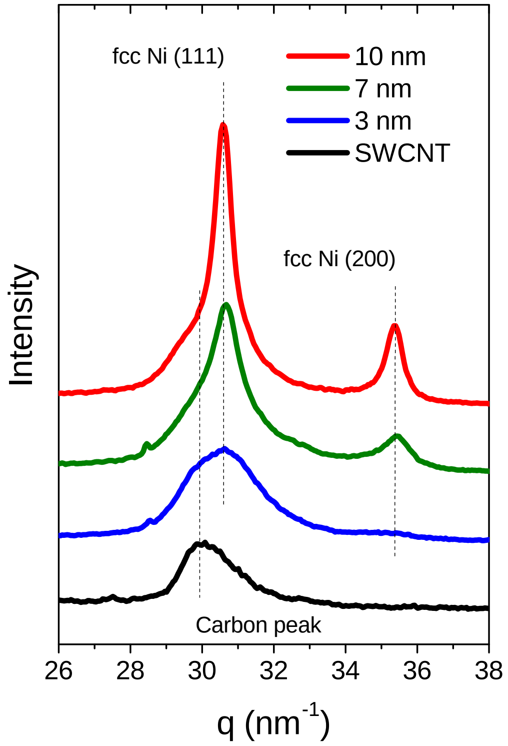

Figure S3: *X-ray diffraction line profiles for the 3, 7 and 10 nm clusters in SWCNTs.*

Raman spectroscopy

A proof of metal clusters encapsulated in SWCNTs on a bulk scale was obtained by checking the filling of SWCNTs with Ni(II) acetylacetonate precursor molecules. It is known that upon heating in vacuum the molecules in SWCNTs are transformed into metal clusters that then act as a catalyst for the formation of inner-shell carbon nanotubes. The Raman spectra for the three samples prepared in the present study exhibit the radial breathing modes (RBMs) of inner tubes after annealing, Fig. S5. The data proves the filling of the SWCNT material with the molecules on a bulk scale, hence the filling with nickel clusters.

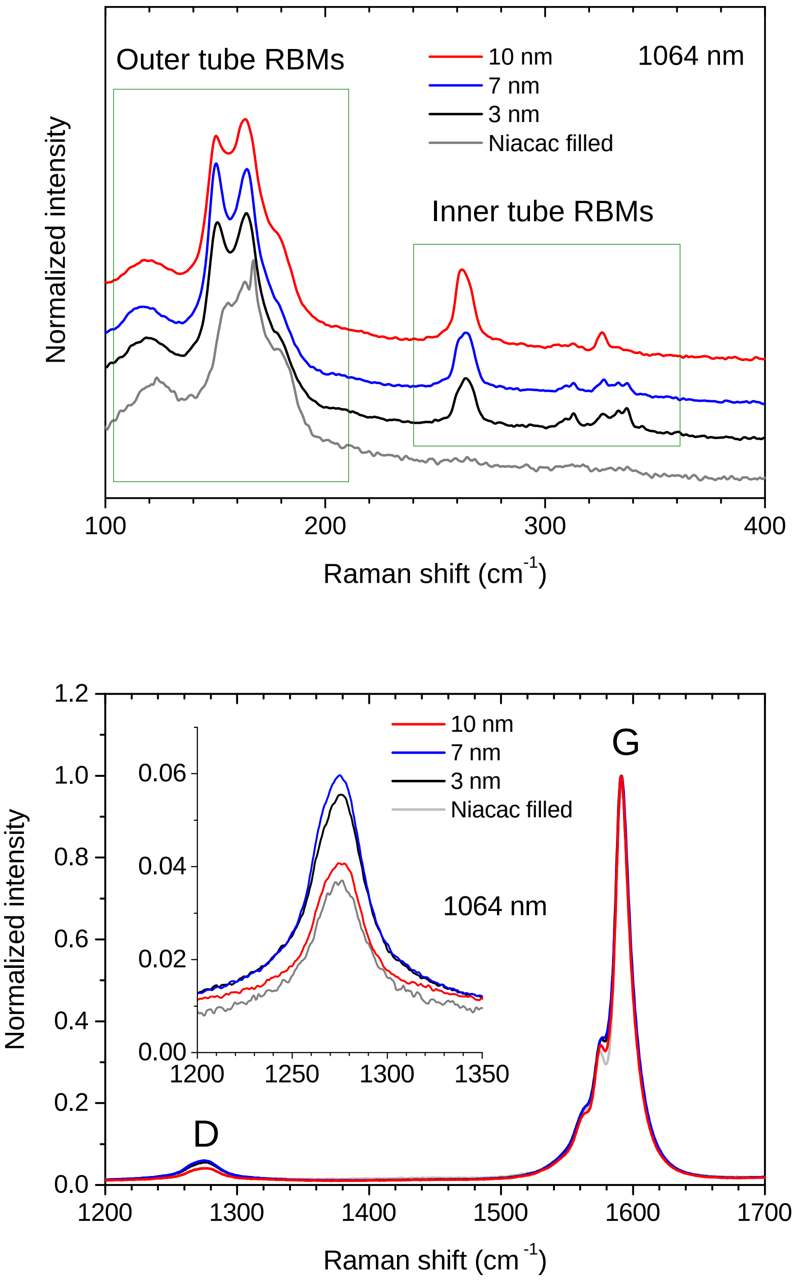

Figure S4: *FT Raman spectra for nickel acetylacetonate-filled SWCNTs before and after being annealed at 500°C for 2 hours, 700°C for 2 hours and 800°C for 5 hours. RBM lines (top) and D and G lines (bottom).*

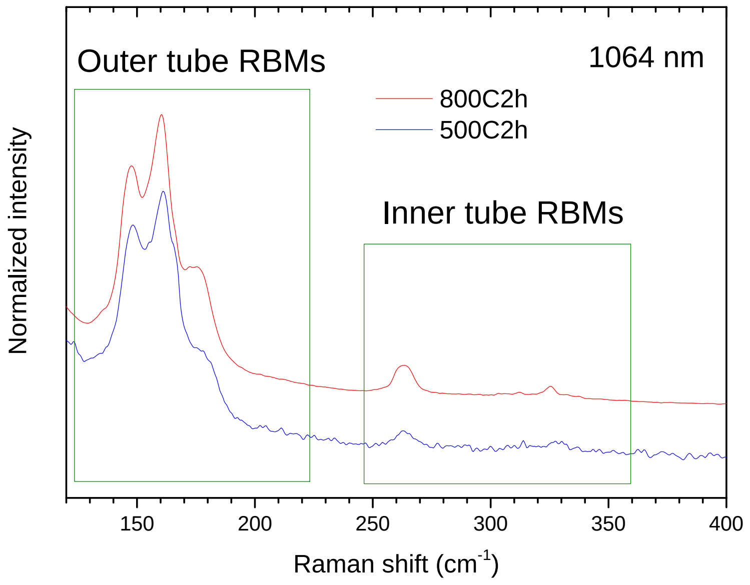

Figure S5: *FT Raman spectra for the nickel acetylacetonate-filled SWCNT sample annealed at 500 °C for 2 hours, on which the transport measurements were carried out, and that annealed at 800 °C for 2 hours. From a comparison of the inner tube RBM line intensities, the filling is about 43% of that for the sample measured by XMCD, SQUID and XRD.*

X-ray photoemission spectroscopy

An elemental analysis was carried out on another nickel acetylacetonate-filled SWCNT sample by means of X-ray photoemission spectroscopy (XPS). Fig. S6 shows the XPS spectra of empty SWCNT, nickel acetylacetonate-filled SWCNTs before and after annealing at 500 °C for 2 hours and 700 °C for 2 hours. The empty SWCNT has no impurities but only 1.8 at.% of oxygen. The nickel $2p$ and oxygen $1s$ lines are observed after the filling. Before annealing, the oxygen-to-nickel ratio is ~ 4.36, consistent with 4 expected for Ni(II) acetylacetonate, molecular formula $C_{10}H_{14}NiO_4$, increased to 11.2 after annealing at 500 °C, and further to 10.5 after annealing at 700 °C.

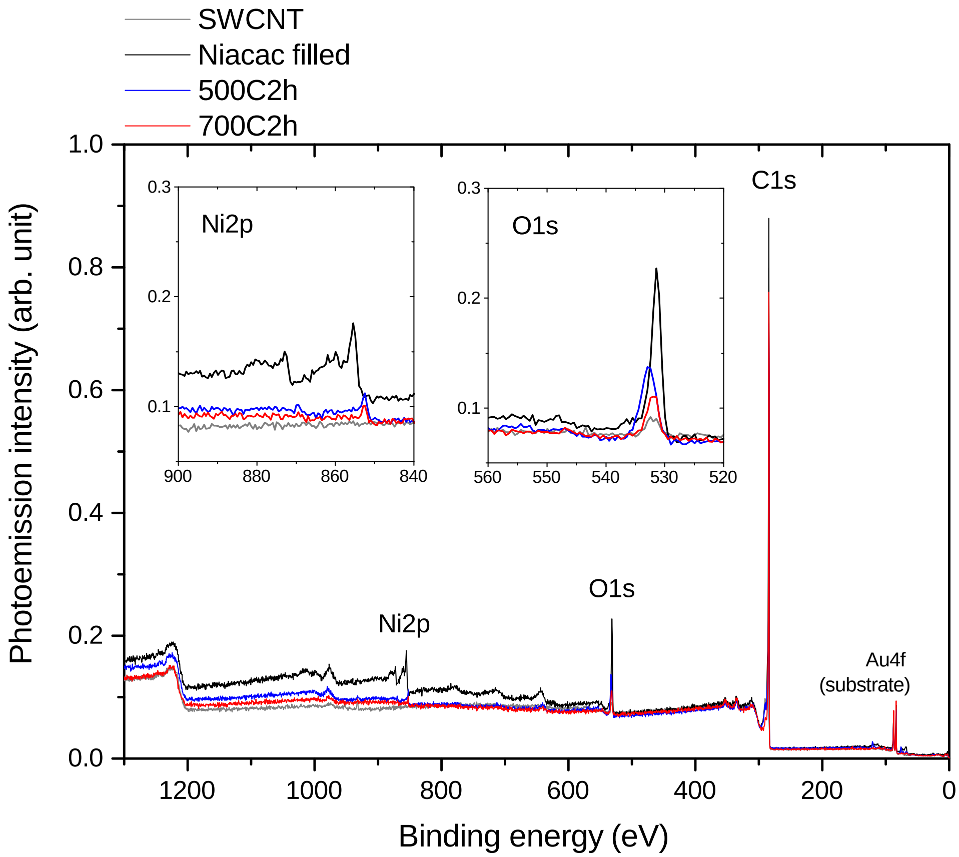

Figure S6: *X-ray photoemission spectra of empty SWCNT, nickel acetylacetonate-filled SWCNTs before and after annealing at 500 °C for 2 hours and 800 °C for 2 hours.*

References

1. Richardson, J. T., Scates, R. & Twigg, M. V. X-ray diffraction study of nickel oxide reduction by hydrogen. *Applied Catalysis A-general* **246**, 137–150 (2003).

2. Langford, J. I. & Wilson, A. J. C. Scherrer after sixty years: A survey and some new results in the determination of crystallite size. *Journal of Applied Crystallography* **11**, 102–13 (1978).

3. Smilgies, D. M. Scherrer grain-size analysis adapted to grazing-incidence scattering with area detectors. *Journal of Applied Crystallography* **42**, 1030–1034 (2009).
